# Complete chloroplast genome of *Cerasus fengyangshanica* (Rosaceae), a wild flowering cherry endemic to China

**DOI:** 10.1080/23802359.2020.1860723

**Published:** 2021-01-21

**Authors:** Xiao-Yan Wang, Ling-Juan Liu, Sheng-Long Liu, Li-Xin Ye, Xue-Kai He, Zhong-Shuai Sun

**Affiliations:** a Zhejiang Provincial Key Laboratory of Plant Evolutionary Ecology and Conservation, Taizhou University, Taizhou, China; b Administration of Fengyangshan-Baishanzu National Natural Reserve, Longquan, China

**Keywords:** *Cerasus fengyangshanica*, flowering cherry, chloroplast genome, phylogenomics

## Abstract

*Cerasus fengyangshanica* is a wild flowering cherry endemic to Mount Fengyang, China. Here, we reported the complete chloroplast (cp) genome of *C. fengyangshanica* (GenBank accession number: MW160272). The cp genome was 157,964 bp long, with a large single-copy region (LSC) of 85,972 bp and a small single-copy region (SSC) of 19,086 bp separated by a pair of inverted repeats (IRs) of 26,453 bp. It encodes 129 genes, including 84 protein-coding genes, 37 tRNA genes, and 8 ribosomal RNA genes. We also reconstructed the phylogeny of *Prunus sensu lato* using maximum likelihood (ML) method, including our data and previously reported cp genomes of related taxa. The phylogenetic analysis indicated that *C. fengyangshanica* is closely related with *Prunus maximowiczii*.

*Cerasus fengyangshanica* L. X. Ye & X. F. Jin is a recently described species, from Mount Fengyang in Zhejiang province, China (Ye et al. 2017). This species was previously treated as *Prunus maximowiczii* in Flora of Zhejiang, but differs in having leaf blades subcapitate-glandulose at margin, bracts clavate-glandulose at margin, stamens 30–32, and bud scales glabrous (Wei and Zhang 1993; Ye et al. 2017). Although it can be distinguished easily from its related species by morphology and geographic distribution, the genetic relationship of *C. fengyangshanica* relative to other flowering cherries has not been well established. According to recent researches, *Cerasus* is a subgenus in *Prunus sensu lato* (Shi et al. 2013; Chin et al. 2014). In this article, we use genus *Prunus* except for *C. fengyangshanica* L. X. Ye & X. F. Jin to prevent confusion. By taking advantages of next-generation sequencing technologies that efficiently provide the chloroplast (cp) genomic resources of our interested species, we can rapidly access the abundant genetic information for phylogenetic research and conservation genetics (Li et al. 2017; Liu et al. 2017). Therefore, we sequenced the whole chloroplast genome of *C. fengyangshanica* to elucidate its phylogenetic relationship with other species in *Prunus sensu lato*.

Total genomic DNA was extracted from silica-dried leaves collected from Fengyangshan-Baishanzu National Natural Reserve (Longquan, Zhejiang province, China) using a modified CTAB method (Doyle and Doyle 1987). A voucher specimen (sun2009001) was collected and deposited in the Herbarium of Taizhou University. DNA libraries preparation and pair-end reads sequencing were performed on the Illumina NovaSeq 6000 platform. The cp genome was assembled via NOVOPlasty (Dierckxsens et al. 2017), using the *Prunus rufa* cp genome (MN648456; Li et al. 2020) as a reference. Gene annotation was performed via the online program Dual Organellar Genome Annotator (DOGMA; Wyman et al. 2004). Geneious R11 (Biomatters Ltd., Auckland, New Zealand) was used for inspecting the cp genome structure.

The complete cp genome of *C. fengyangshanica* (GenBank accession number: MW160272) was 157,964 bp long consisting of a pair of inverted repeat regions (IRs with 26,453 bp) divided by two single-copy regions (LSC with 85,972 bp; SSC with 19,086 bp). The overall GC contents of the total length, LSC, SSC, and IR regions were 36.7%, 34.6%, 30.2% and 42.5%, respectively. The genome contained a total of 129 genes, including 84 protein-coding genes, 37 tRNA genes and 8 rRNA genes.

We used a total of 22 additional complete cp genomes of the *Prunus sensu lato* species to clarify the phylogenetic position of *C. fengyangshanica*. *Prunus serotina* Ehrh. (NC036133) and *P. padus* L. (NC026982) in Subg. *Padus* were used as the outgroup. We reconstructed a phylogeny employing the GTR + G model and 1000 bootstrap replicates under the maximum-likelihood (ML) inference in RAxML-HPC v.8.2.10 on the CIPRES cluster (Miller et al. 2010). The ML tree (Figure 1) was consistent with the most recent phylogenetic study on *Prunus sensu lato* (Shi et al. 2013; Chin et al. 2014), but inconsistent with the phylogenetic result when it was published (Ye et al. 2017). *Cerasus fengyangshanica* exhibited the closest relationship with *Prunus maximowiczii.*

**Figure 1.**
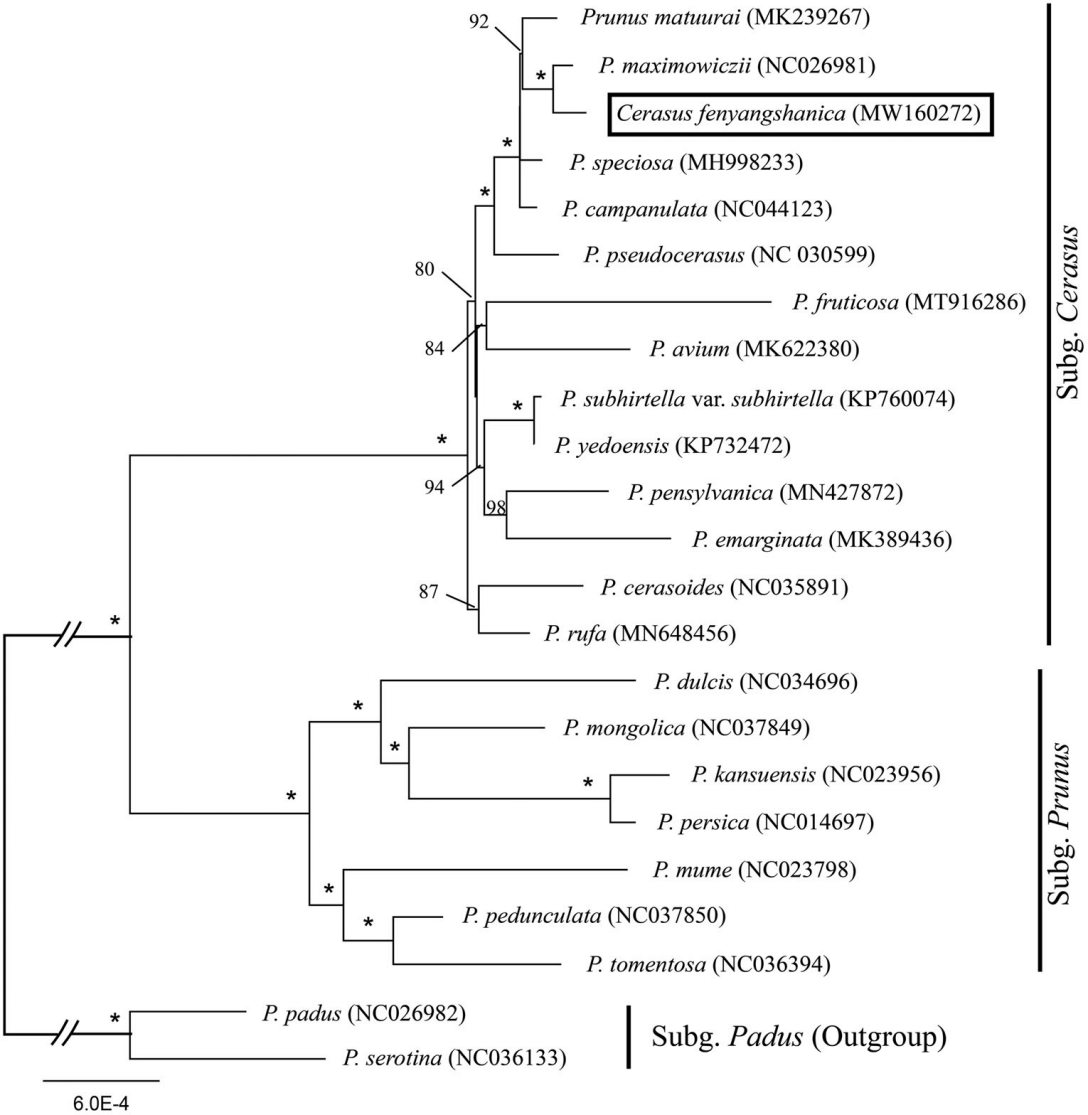
Phylogenetic tree reconstruction of 23 taxa of *Prunus sensu lato* using ML method. Relative branch lengths are indicated. Support values above the branches are ML bootstrap support; ‘*’ indicates 100% support values.

## Data Availability

The genome sequence data that support the findings of this study are openly available in GenBank of NCBI at (https://www.ncbi.nlm.nih.gov/) under the accession no. MW160272. The associated BioProject, SRA and BioSample numbers are PRJNA678521, SRR13067457 and SAMN16803144 respectively.
